# Association between COVID-19 Vaccination and SARS-CoV-2 Infection among Household Contacts of Infected Individuals: A Prospective Household Study in England

**DOI:** 10.3390/vaccines12020113

**Published:** 2024-01-23

**Authors:** Khitam Muhsen, Pauline A. Waight, Freja Kirsebom, Nick Andrews, Louise Letley, Charlotte M. Gower, Catriona Skarnes, Catherine Quinot, Rachel Lunt, Jamie Lopez Bernal, Stefan Flasche, Elizabeth Miller

**Affiliations:** 1 Department of Epidemiology and Preventive Medicine, School of Public Health, Faculty of Medicine, Tel Aviv University, Tel Aviv 6139001, Israel; 2 London School of Hygiene and Tropical Medicine, London WC1E 7HT, UK; stefan.flasche@lshtm.ac.uk (S.F.); liz.miller@lshtm.ac.uk (E.M.); 3 UK Health Security Agency, 61 Colindale Avenue, London NW9 5EU, UK; paulinekaye@sky.com (P.A.W.); freja.kirsebom@ukhsa.gov.uk (F.K.); nick.andrews@ukhsa.gov.uk (N.A.); catrionaskarnes95@gmail.com (C.S.); jamie.lopezbernal2@ukhsa.gov.uk (J.L.B.); 4 NIHR Health Protection Research Unit in Respiratory Infections, Imperial College London, London SW7 2AZ, UK

**Keywords:** COVID-19 vaccination, household transmission, alpha variant, delta variant, omicron variant

## Abstract

Background: We investigated whether COVID-19 vaccination reduced SARS-CoV-2 infection risk among adult household contacts of COVID-19 index cases during the Alpha, Delta, and Omicron waves in England. Methods: Between February 2021 and February 2022, SARS-CoV-2 RT-PCR nasal swabs were collected from COVID-19-confirmed index cases aged ≥20 years and their household contacts at enrolment and three and seven days thereafter. Generalized Estimating Equations models were fitted with SARS-CoV-2 positivity as the outcome and household contacts’ vaccination status as the main exposure while adjusting for confounders. Results: SARS-CoV-2 infection was confirmed in 238/472 household contacts (50.4%) aged ≥20 years. The adjusted relative risk (95% confidence interval) of infection in vaccinated versus unvaccinated household contacts was 0.50 (0.35–0.72) and 0.69 (0.53–0.90) for receipt of two doses 8–90 and >90 days ago, respectively, and 0.34 (0.23–0.50) for vaccination with three doses 8–151 days ago. Primary vaccination protected household contacts against infection during the Alpha and Delta waves, but only three doses protected during the Omicron wave. Vaccination with three doses in the index case independently reduced contacts’ infection risk: 0.45 (0.23–0.89). Conclusions: Vaccination of household contacts reduces their risk of infection under conditions of household exposure though, for Omicron, only after a booster dose.

## 1. Introduction

The extensive investment and rapid development and deployment of COVID-19 vaccines were essential in controlling the COVID-19 pandemic [1,2,3,4]. In the United Kingdom (UK), vaccination with the BNT162b2 or the ChAdOx1-S vaccines conferred protection against COVID-19 and related hospitalization in older adults during the predominance of the Alpha variant (B.1.1.7) [5], as well as vaccination with two doses during the Delta variant (B.1.617.2) predominance [6]. Vaccine effectiveness decreased 20 weeks post-vaccination with the second dose [7], suggesting waning vaccine-induced immunity [8,9,10,11]. Israel was the first country to introduce booster vaccination using the BNT162b2 mRNA vaccine in July 2021 to address waning immunity and control the Delta variant surge, a policy that was highly effective against COVID-19 hospitalizations and deaths [12,13] and was adopted widely by other countries using mRNA vaccines.

The emergence of the highly transmissible Omicron variant (B.1.1.529) in November 2021, with its numerous mutations in the Spike encoding genes and immune escape abilities [14,15,16], led to a marked increase in COVID-19 incidence. In response to Omicron emergence, Israel introduced the second booster (fourth dose) of the BNT162b2 vaccine in December 2021 to enhance existing immunity, provide cross-protection and prevent severe COVID-19 [17] a policy that was followed in other countries thereafter. COVID-19 booster vaccination was introduced in the UK in September 2021 for all adults aged 50 years and over, health and social care workers, and those in clinical risk groups six months after completing the primary series, with an additional booster recommended in the spring of 2022 [18]. In the UK, the administration of a booster dose of mRNA vaccines enhanced protection against COVID-19 caused by the Omicron variant [19]. The UK COVID-19 booster vaccination policy was expanded from age 40+ years to adults aged 18–39 years in light of the emergence of the Omicron variant [20]. While abundant evidence exists on COVID-19 vaccine effectiveness at the individual level, gaps remain in the knowledge regarding the effect of COVID-19 vaccination on the spread of SARS-CoV-2 infections within households with COVID-19 cases [21,22,23,24,25,26]. Household studies are critical given the high risk of SARS-CoV-2 transmission in these settings, as we and others have shown [25,27,28,29,30,31,32,33,34,35,36,37]. Prior household studies showed a reduced risk of SARS-CoV-2 infection in vaccinated versus unvaccinated household contacts with an infected household member [21,23,24,38,39,40,41,42,43]. Despite this evidence, gaps in knowledge remain mainly because these studies were conducted shortly after the rollout of COVID-19 vaccines during the periods of the Alpha or Delta variants predominance and before complex issues in COVID-19 vaccination have emerged, such as waning immunity and booster vaccination. Accordingly, we examined the association between COVID-19 vaccination and the risk of SARS-CoV-2 infection among household contacts of confirmed cases in a prospective household study between February 2021 and February 2022 in England. This study period captured the Alpha, Delta, and Omicron waves and the rollout of the first booster vaccinations in England. We hypothesized that COVID-19 vaccination would be inversely associated with SARS-CoV-2 infection in household contacts of infected individuals. We also explored the association of the index cases’ vaccination with infection among the household contacts and persistent SARS-CoV-2 positivity among the index cases.

## 2. Materials and Methods

### 2.1. Study Design and Population

Following the introduction of COVID-19 vaccination, the UK Health Security Agency (UKHSA) set up enhanced surveillance to understand how vaccination impacted individuals’ response to COVID-19 infection. As previously described, SARS-CoV-2 RT-PCR positive cases in the community who were tested because of symptoms, or in a minority because of a known contact, were prospectively followed up in England between February 2021 and February 2022 with sequential naso/pharyngeal swabs and a convalescent blood sample to document viral kinetics and serological responses [44]. Individuals were not eligible for inclusion in the enhanced surveillance if they were under 18 years of age, enrolled in other studies, residents of a care home, had a previous positive PCR test within 90 days, or tested positive more than 10 days post-symptom onset [44]. Cases with a least one household member without a prior SARS-CoV-2 positive test were invited to take part in a nested prospective household contact study to document the effect of vaccination on transmission [40]. Index cases and their household contacts (one or more of any age) who provided informed verbal consent were interviewed via telephone at recruitment by UKHSA study nurses to collect information on demographics and the following solicited symptoms (fever, cough, loss of smell/taste, runny nose, sore throat, nausea, diarrhea, headache, muscle/body pain, fatigue), symptom onset dates, and COVID-19 vaccination status (number of doses, vaccination date, and vaccine type). Information on symptoms was also collected 21 days thereafter. The status of the index case or household contact was defined based on the index case who was the first laboratory-confirmed index case among the household members and identified via the national PCR testing system, regardless of the symptom onset date reported by infected household contacts, since symptoms can be unrelated to COVID-19 [28]. The reported COVID-19 vaccination status was validated using the National Immunisation Management System.

Packs containing virus transport medium and swabs were couriered to the study households to obtain self-taken nasal swab specimens on recruitment and three and seven days later (follow-up nasal swabs). We included in the analysis participants who provided at least one swab. Nasal swabs were tested at UKHSA Virus Reference Department at Colindale using dual target RT-PCR (ORF and E genes). Individuals who did not provide any swabs were excluded from the analysis.

### 2.2. Study Variables

The primary analysis in this study focused on the association between the household contacts’ vaccination status and overall SARS-CoV-2 infection in the contacts.

1. The primary outcome variable was RT-PCR-confirmed SARS-CoV-2 infection (yes or no) in at least one of the follow-up nasal swabs, hereafter referred to as SARS-CoV-2 infection. A positive SARS-CoV-2 RT-PCR result was defined as cycle threshold (Ct) values ≤39 [28]. Secondary outcomes included confirmed SARS-CoV-2 infection (yes or no) in each swab, positive in all swabs or negative in all swabs. The persistence of SARS-CoV-2 positivity among the index cases was also examined and defined as a positive RT-PCR SARS-CoV-2 in the follow-up-swabs. In an exploratory analysis, the Ct value of the first positive RT-PCR test result of the follow-up swabs of the index cases and household contacts was analyzed as an outcome variable, categorized by the median level.

2. The main exposure variable in the primary analysis was COVID-19 vaccination status of the household contacts. In additional analyses, the index cases’ vaccination status was also examined. Vaccination status was defined as follows:

(1) The number of vaccine doses received (not vaccinated, one, two, or three doses).
(2) The number of vaccine doses and type received: the ChAdOx1-S adenovirus vector vaccine (Oxford-AstraZeneca, AstraZeneca AB, Sodertalje, Sweden) COVID-19 ChAdOx1-S vaccine, mRNA-1273 (Moderna, Inc., Cambridge, MA, USA) and BNT162b2 (Pfizer-BioNTech, Marburg, Germany) vaccines. Since only a few participants received the mRNA-1273 vaccine, we grouped mRNA-1273 and BNT162b2 vaccines into one category of mRNA vaccines.
(3) The number of vaccine doses received and time elapsed since the last dose were grouped as follows: (a) unvaccinated and vaccinated with one dose ≤ 21 days (as no protection is expected before 21 days post-vaccination with the first dose) [45] (b) one dose > 21 days and two doses ≤ seven days (as protection is anticipated > seven days-post vaccination); (c) two doses 8–90 days; (d) two doses > 90 days and three doses ≤ seven days (as the booster impact is anticipated > seven days-post vaccination with the second dose), and (e) three doses 8–151 days. COVID-19 booster vaccination was introduced in the UK in September 2021, and the time elapsed since vaccination with the third dose in this study ranged between 0 and 151 days. The number of participants who received three doses > 90 days ago was small (n = 9); thus, recipients of the third dose were grouped into one category of 8–151 days post-vaccination.

For the household contacts (primary analysis), the time difference was calculated as the interval between the date of symptom onset and the vaccination date. If they reported no symptoms, the date of the first positive RT-PCR test was used. For household contacts with negative follow-up RT-PCR test results, the date of providing the last nasal swab was used. This approach captures changes in immunity over time since vaccination, both the development and waning immunity [9,45,46,47]. The time elapsed since vaccination for the index case (secondary analyses) was calculated as the difference between the date of symptom onset (or date of the first positive RT-PCR results for asymptomatic index cases) and the vaccination date.

3. Covariates: age in years (1–4, 5–15, 16–19, 20–29, 30–39, 40–49, 50–59, 60–87), sex, household size including children and adults (small households [two or three individuals] or larger households [four to eight] individuals) and variant (study) period, which was determined using the enrolment date. Following the UKHSA published data on SARS-CoV-2 variants circulating in England [48], weeks 6–18 of 2021 were considered as the Alpha variant predominance period, weeks 19–48 of 2021 Delta variant predominance period, and weeks 49–52 of 2021 and weeks 1–6 of 2022 were considered Omicron variant predominance period. The predominance period for a variant was defined as the period in which ≥50% of the sequenced samples consisted of that variant. Since there were overlapping periods, during which different variants were co-circulating, the variable time was analyzed using small width categories of calendar month and year to account for changes over time. In an additional model vaccination status of the index cases (as defined above) was analyzed as an independent variable.

### 2.3. Data Analysis

Characteristics of the participants were described using median and interquartile range (IQR) for continuous and discrete variables and numbers and percentages for dichotomous and categorical variables. COVID-19 vaccination status was described according to demographics and study period. The numbers and percentages of participants with SARS-CoV-2 infection in the follow-up swabs were described according to demographics, study period, and COVID-19 vaccination status, separately for the index cases and household contacts. We fitted Generalized Estimating Equations (GEE) for each group with robust variance estimates to account for the clustering within households [49], similar to the approach used in other COVID-19 household studies [50,51]. We used negative binomial models with log link to examine the associations of COVID-19 vaccination and the covariates mentioned above, with SARS-CoV-2 infection. Relative risks (RR) and the 95% confidence intervals (CI) were obtained from these models. The negative binomial models were used since the outcome variable was common [52].

Ct values of the first positive SARS-CoV-2 result in the follow-up swabs were categorized by the median and compared by the number of COVID-19 vaccine doses received by the participant. Using GEE, we examined the association between vaccination and Ct value as a proxy for viral load [53]. Higher Ct values represent lower viral load.

The following sensitivity analyses were undertaken: (1) analysis of the association between COVID-19 vaccination and symptomatic SARS-CoV-2 infection among the household contacts while excluding those with asymptomatic infection from the analysis; (2) using calendar month/year at enrolment to adjust for variant/period; and (3) a stratified analysis by variant/period (supplementary methods). We assessed the correlation between vaccination status (number of doses) of the index case and that of the contact using Spearman’s correlation coefficient and using GEE we explored the potential role of COVID-19 vaccination in the index case on the risk of infection in the household contact beyond the contact’s vaccination status.

Data were analyzed using IBM SPSS version 28 (IBM, Armonk, New York, NY, USA).

### 2.4. Ethics Approval

The UKHSA Research Ethics and Governance Group approved the household surveillance protocol as part of the UKHSA enhanced surveillance in response to the COVID-19 pandemic. Verbal informed consent was obtained from adults, and oral consent was obtained from the legal guardian for children. Herein only anonymized data were used.

## 3. Results

**Figure 1:**
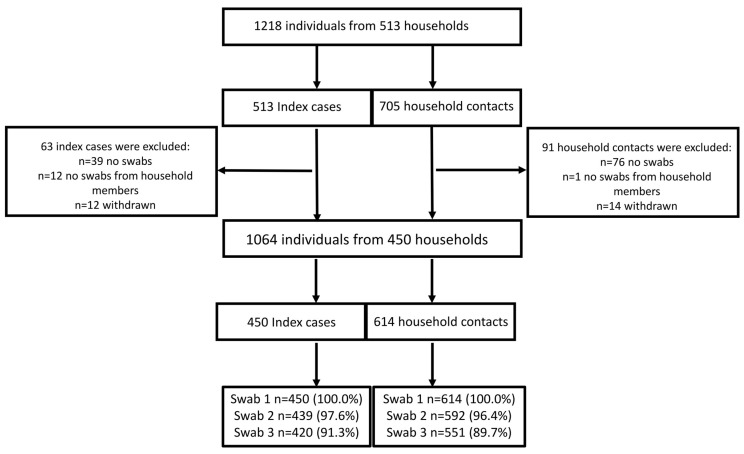
Flow chart.

Overall, 1218 individuals from 513 households, 513 index cases, and 705 household contacts were enrolled between February 2021 and February 2022. The 512 index cases comprised 33.6% of the 1526 cases included in the main enhanced surveillance study who met the inclusion criteria [44]. A total of 154 individuals were excluded (115 did not provide any swabs, 13 individuals provided swabs, but their household members did not, and 26 withdrew), thus leaving in the analysis 1064 individuals comprising 450 index cases and 614 household contacts who provided at least one nasal swab. Second and third nasal RT-PCR swabs were provided by 96.9% and 91.3% of the participants (Figure 1).

The characteristics of individuals included and those excluded from the study were similar, except that the latter had proportionately more unvaccinated individuals and proportionately fewer vaccinees with three doses (Supplementary Table S1). The median number of participants per household was two (range 2 to 6). Most participants (89.4%) were recruited within seven days of symptoms onset in the index cases. Participants were enrolled during periods with different predominant SARS-CoV-2 variants (Supplementary Figure S1), with 28.1%, 57.0%, and 14.8% of the participants enrolled during the Alpha, Delta, and Omicron predominance periods, respectively. The age ranged from 21 to 87 years (median = 50) in the index cases and from one to 87 years (median = 42) in the household contacts. The respective proportions of males were 39.1% and 52.3%. Most participants were of white ethnicity (Table 1).

One or more solicited symptoms were reported by 94.9% of index cases and 96.2% of household contacts aged ≥20 years who had RT-PCR evidence of infection (238/472, 50.4%). Cough, fever, and loss of taste/smell were reported among 74.2%, 53.6%, and 50.4%, respectively, of the index cases, and 93.3% had other symptoms (Supplementary Table S2).

### 3.1. COVID-19 Vaccine Uptake

Among the index cases, 18.9% received one dose of a COVID-19 vaccine, 44.9% and 20.9% received two and three doses, respectively, while 15.3% were unvaccinated. Index cases aged 20–29 and 30–39 years were more often unvaccinated or vaccinated with one dose, while older index cases were more often vaccinated with two or three doses. Individuals enrolled during the Alpha period were more frequently unvaccinated or vaccinated with one dose, while those enrolled during the Delta and Omicron periods were more frequently vaccinated with two or three doses (Table 2).

Among all household contacts, 34.4% were unvaccinated, 15.5%, 37.0%, and 13.0% received one, two, and three doses of a COVID-19 vaccine, respectively. One (0.1%) contact received four doses and was included in the three-dose group. Individuals aged under 20 years comprised over 50% of unvaccinated household contacts, 13.7% and 4.4% of those vaccinated with one and two doses, respectively, but none of this age group was vaccinated with a third dose, while the proportion of older age groups was higher among the vaccinated household contacts (Supplementary Table S3); this is consistent with the delayed introduction of COVID-19 vaccines in September 2021 for healthy children aged 12–15 years and later for younger ages. Therefore, we limited the analysis on COVID-19 vaccination to household contacts aged 20 years or older (i.e., excluding those aged < 20 years, while keeping households with older household contacts). In this group 19.7%, 17.4%, 40.6%, and 16.9% of the participants were unvaccinated, vaccinated with one, two, and three doses, respectively (Supplementary Table S4). The time elapsed since vaccination with the third dose among the study participants ranged between 0 and 151 days. Participants aged 20–29 and 30–39 were more often unvaccinated against COVID-19, while older household contacts received two or three doses more frequently. The proportion of females was higher in the vaccinated than unvaccinated household contacts. COVID-19 vaccination status differed according to the study period also in the contacts (Table 2).

The type of COVID-19 vaccines and the time elapsed since vaccination were comparable between the index cases and household contacts (Supplementary Table S4).

### 3.2. SARS-CoV-2 Follow-Up Nasal Swabs among the Index Cases

A median of six days (IQR = 3) elapsed between the onset of symptoms in the index cases and providing the first follow-up nasal RT-PCR swab, and nine days (IQR = 3), and 13 (IQR = 3) for the second and third swabs, respectively. Overall, 398 index cases (88.4%) tested positive in at least one follow-up swab, with decreased positivity from 86.9% in the first swab to 43.1% in the third swab. The likelihood of SARS-CoV-2 positivity in the follow-up swabs was inversely related to vaccination with three doses. The results were consistent when considering secondary outcomes, such as testing positive or negative in all three follow-up swabs (Supplementary Table S5A–C).

### 3.3. SARS-CoV-2 Infection among Household Contacts Aged 20 Years or Older

The SARS-CoV-2 infection rate was lower among household contacts aged under 30 years compared to older ones (Table 3). The infection rate was highest (59.1%) among household contacts who did not receive a COVID-19 vaccine and lower among recipients of three doses (41.3%). The proportion of household contacts with SARS-CoV-2 infection was lower among recipients of an mRNA COVID-19 vaccine than unvaccinated ones. Household contacts who were vaccinated with sufficient time to generate protection against COVID-19 from that dose (one dose > 21 days, two doses 8–90 days, and three doses 8–151 days) had a lower risk of infection than unvaccinated participants (*p* < 0.001) (Table 3). Findings were consistent considering secondary outcomes (Supplementary Table S6A,B).

A multivariable model showed a lower risk of SARS-CoV-2 infection among recipients of two doses: RR = 0.66 (95% CI 0.50–0.86) and three doses of a COVID-19 vaccine: RR = 0.40 (95% CI 0.28–0.59) than unvaccinated ones (Table 3). This model showed an increased risk of SARS-CoV-2 infection with age by 1.36–1.78-fold (*p* = 0.006), and during the periods in which the Delta (RR = 1.36 (95% CI 1.04–1.79)) and Omicron variants (RR = 2.01 (95% CI 1.41–2.86)) were predominant versus the period in which the Alpha variant was dominant. However, the risk decreased in household contacts from larger versus smaller households (RR = 0.81 (95% CI 0.66–0.99)). Another model showed a significantly lower risk of SARS-CoV-2 infection among household contacts who received one (*p* = 0.040), two (*p* < 0.001), or three doses of an mRNA vaccine (*p* < 0.001), although the association was stronger for receiving three doses. Vaccination with primary series with ChAdOx1-S vaccine and a booster dose with an mRNA vaccine was also associated with a lower risk of infection (*p* < 0.001). When considering the time since last dose, a lower risk of infection was found >21 days after vaccination with the first dose (RR = 0.72 (95% CI 0.52–0.98), within 8–90 days of a second dose, the risk was also lower (RR = 0.50 (95% CI 0.35–0.72)) than for those vaccinated after 90 days (RR = 0.69 (95% CI 0.53–0.90), with the lowest risk (RR = 0.34 (95% CI 0.23–0.50)) found for vaccination with three doses 8–151 days ago (Table 3).

### 3.4. Sensitivity Analyses

Limiting the analysis to individuals with symptomatic infection showed similar results (Supplementary Table S7). Using the variable calendar month/year to adjust for variant/period showed slightly stronger associations between COVID-19 vaccination and overall SARS-CoV-2 infection and symptomatic infection (Supplementary Table S8).

### 3.5. Stratified Analysis by Variant/Period

COVID-19 vaccination status differed across the study periods (Supplementary Table S9), making it difficult to directly compare the protection afforded by the same vaccination schedule against the different variants. During the Alpha variant period, vaccination with one or two doses was inversely associated with SARS-CoV-2 infection among the household contacts: adjusted RR = 0.59 (95% CI 0.37–0.93); the respective estimates for vaccination with one dose > 21 days ago and two doses within the past 8–90 days were 0.44 (0.27–0.73) and 0.28 (0.08–0.99) (Supplementary Table S10).

The corresponding estimates during the Delta wave were 0.56 (0.36–0.87), and 0.17 (0.07–0.40), for receiving two and three doses, and 0.10 (0.03–0.32) for receiving three doses in the preceding 8–151 days (Supplementary Table S11).

During the Omicron wave, the association of vaccination with three doses 8–151 days ago and infection was attenuated: RR = 0.65 (0.42–0.99) (Supplementary Table S12).

### 3.6. The Association between COVID-19 Vaccination Status of the Index Case and Risk of Infection among the Household Contacts

COVID-19 vaccination status of the index cases and that of the household contacts were strongly correlated (Supplementary Table S13): Spearman’s correlation coefficient 0.72, *p* < 0.001. Bivariate analysis showed a lower risk of infection in the household contacts if the index case in their household was vaccinated with three doses versus unvaccinated index cases, unadjusted RR = 0.67 (95% CI 0.48–0.93), *p* = 0.018. This association remained significant in a multivariable model that included the household contacts’ vaccination status, adjusted RR = 0.63 (95% CI 0.40–0.98), *p* = 0.045. Restricting this analysis to the Delta wave, during which the booster vaccination began, demonstrated a stronger association: adjusted RR = 0.45 (0.23–0.89) *p* = 0.021 (Table 4). The number of participants was small for a separate analysis of the Omicron period.

### 3.7. COVID-19 Vaccination and SARS-CoV-2 Ct Values

The proportion of individuals with high Ct values of the ORF gene in SARS-CoV-2 positive index cases and household contacts was highest among vaccinees with three doses of a COVID-19 vaccine, who were 12% more likely to have high Ct values than unvaccinated participants (Table 5). The results were similar for the E gene (Supplementary Table S14).

## 4. Discussion

We confirmed our main hypothesis of an association of COVID-19 vaccination and reduced risk of SARS-CoV-2 infection among household contacts of COVID-19 index cases. Our study spanned over 12 months between February 2021 and February 2022, while COVID-19 vaccination policy was evolving globally and covered the Alpha, Delta, and Omicron variants waves. During this period, major insights were gained regarding COVID-19 vaccinology, including the waning of vaccine-induced immunity [7,8,9,10] and the need for booster vaccination to overcome waning immunity and provide cross-protection against emerging variants [11,17,19].

We found that household contacts who received two doses of a COVID-19 vaccine had a statistically significant 34% lower risk of SARS-CoV-2 infection, and for those who received three doses their risk declined by 60% compared to unvaccinated household contacts of COVID-19 index cases. We found that vaccination with one or two doses of mRNA COVID-19 vaccines significantly reduced the risk of infection among the household contacts by 42% and 45%, respectively, while vaccination with two doses of the ChAdOx1-S COVID-19 vaccine (Oxford-AstraZeneca) was associated with a 25% risk reduction. Vaccination with three doses of mRNA vaccines showed a 63% reduced risk of infection, which was also reduced by 54% among household contacts who received a primary series with the ChAdOx1-S and boosted with an mRNA vaccine. The confidence intervals of these estimates were overlapping; therefore, we cannot definitively infer differences by vaccine brand. Importantly, we found that recent COVID-19 vaccination was associated with a significant risk reduction of SARS-CoV-2 infection among the household contacts. The risk was reduced by 50% for vaccination with two doses within 8–90 days; this association was attenuated >90 days post-second dose. The maximum risk reduction of 66% (RR = 0.34) was attained 8–151 days post-vaccination with the third dose. The time elapsed since vaccination with the third dose among the participants ranged between 0 and 151 days, and we could not assess the waning immunity of the third dose, due to small sample size.

Prior household studies showed that fully vaccinated contacts of SARS-CoV-2 confirmed cases had a significantly lower risk of having the infection compared to unvaccinated household contacts; the vaccine effectiveness estimates ranged between 61% and 89% during the Alpha and Delta waves [21,23,24,38,54]. These vaccine effectiveness estimates are lower than in population-based effectiveness studies, which capture protection under a variety of exposures and, in some instances, have focused on hospitalization and death as the outcome [6,19,55]. Our point estimates were slightly lower than reported in prior household studies [21,23,24,38,54], likely due to the inclusion of infections caused by the Omicron variant in our study in contrast to previous studies that captured mainly the Alpha and Delta variants waves.

Evidence regarding COVID-19 vaccine effectiveness in household contacts according to SARS-CoV-2 variants is inconsistent. A study from Israel [24] showed reduced vaccine effectiveness among household contacts during the Delta versus the Alpha wave, while a study from Spain did not demonstrate such differences [38]. While in our study, we could not show differences in vaccine effect on household transmission between the Alpha and Delta variants, it was clear that vaccination reduced the risk of transmission in household settings in both periods and that recently vaccinated household contacts with three doses in the preceding 8–151 days had the highest protection from infections during the Delta wave. Our study suggests this protective effect was substantially attenuated during the Omicron wave, likely due to the immune-escape ability of Omicron [14,16] and higher transmission that was also shown in household settings [35,36,41]. These results show the cross-protection attained by recent booster vaccination with the Wuhan strain against emerging variants. A meta-analysis of household studies showed that complete vaccination against COVID-19 was associated with higher protection against the Alpha variant 94.7%, which was reduced to 64.4% for Delta, and 35.8% for Omicron [56]. Collectively our and others’ findings indicate that COVID-19 vaccination reduces the risk of transmission of SARS-CoV-2 infection to household contacts of COVID-19 cases, regardless of the circulating variant. However, for Omicron, this effect was diminished. While current COVID-19 vaccination policies focus less on the prevention of transmission, reducing the risk of transmission likely remains relevant for healthcare workers and their contacts.

The effect of COVID-19 vaccination on the risk of infection in the household contacts can be attributed to the direct protection conferred by vaccination and likely to indirect protection attained by vaccination of the index cases resulting in reduced transmission of SARS-CoV-2. Index cases vaccinated against COVID-19 were less likely to test positive for SARS-CoV-2 during the follow-up than unvaccinated index cases, suggesting that COVID-19 vaccination shortens the duration of shedding the virus and transmission within households. Moreover, we found a significantly decreased risk of SARS-CoV-2 infection in household contacts whose index cases received three doses (Table 4), independent of the contact’s vaccination status. This finding supports our prior report showing a reduction in the SARS-CoV-2 household transmission in relation to vaccination of index cases, employing a Bayesian approach and data obtained during the Alpha and Delta waves, but not including the Omicron wave [40]. A prior study from England that utilized national surveillance data on laboratory-confirmed COVID-19 cases sharing the same address who passively presented for testing showed a ~2-fold higher secondary attack rate of SARS-CoV-2 infection among unvaccinated household contacts of unvaccinated index cases compared to vaccinated index cases with one dose >21 days during the Alpha variant surge [22]. However, such a difference diminished during the Delta wave, including in relation to vaccination with two doses [39,57]. Another study from England also using national surveillance data showed a-38% reduced risk of SARS-CoV-2 transmission to household contacts during the Delta period in relation to vaccination with three doses in the index cases versus no vaccination; the risk reduction was comparable for household contacts vaccinated with three doses versus unvaccinated household contacts; these associations diminished substantially during the Omicron wave [41], emphasizing the positive impact of the booster dose, as we have shown in this study. The number of participants during the Omicron period in our study was small; thus, we could not robustly assess the role of the booster vaccination in the index cases on the household contacts’ risk of infection during the Omicron era. The lower Ct values of SARS-CoV-2 positive vaccinated individuals compared to unvaccinated ones [58,59] might also suggest lower viral load and thus, less transmission from these cases to their household contacts.

We also found that older household contacts were at higher risk of infection than younger ones, in agreement with prior reports [24,27]. Older household contacts were more often vaccinated, which might have led to less stringent self-isolation measures in these households. We also found a lower risk of infection in household contacts from larger households than those from smaller households, which is consistent with our early reports [27,28] but not with other studies [23,60,61]. While a positive association between household crowding and increased risk of infection [61] is intuitive, the explanation of an inverse association is more challenging and might be related to socioeconomic status, COVID-19 risk perception and behaviors [62], but the exact mechanism remains unclear.

Our study has limitations. About 12% of the enrolled individuals were excluded from the analysis mainly because they did not provide follow-up swabs. The excluded group had a higher proportion of individuals who were unvaccinated against COVID-19 and a lower proportion of vaccinees with three doses. This might have weakened the effect of COVID-19 vaccination in our study, since the attack rate might be larger if those individuals were included. Most (>90%) SARS-CoV-2 infections in our study were symptomatic. We relied on the participants’ reports to gather information on symptoms and onset date, which might be affected by recall bias, as well as the reporting of symptoms not due to COVID-19. Self-reported COVID-19 symptoms of fever, cough, and loss of taste/smell, were shown to have a high positive predictive of value for infection (67%) in our previous study [28] and in the current study one or more of these symptoms was reported by 86.7% and 84.0% of index cases and PCR positive contacts, respectively. Our assumption was that most infections in the household contacts were due to household exposure. While we did not confirm this by sequencing data, a prior analysis from this cohort up to September 2021 (i.e., the Alpha and Delta periods) included whole-genome sequencing of 92 PCR positive index case-household contact pairs from 79 households, with 345 whole-genome sequences (including longitudinal samples), showed that only in two households the phylogeny supported acquisition of the infection outside the household [40]. Household contacts were eligible for inclusion in the study if they have not had a prior SARS-CoV-2, but some individuals might have had undocumented asymptomatic infection. Thus, our analysis does not account for hybrid immunity. The measurements of antibodies against antibodies against the nucleocapsid antigen is another alternative for the detection of SARS-CoV-2 infection, however, serology and oral fluid specimens were available only for a subset of the study participants in our study. The sample frame of the index case was individuals who performed PCR community testing, who might have different characteristics to the general population. However, this should not affect the results of associations between COVID-19 vaccination and the risk of infection among their household contacts. Regarding vaccination status, 84.6% of the index cases in our study received at least one dose of a COVID-19 vaccine, compared to 92.0% in the general population aged 18 and over in the UK as of January 2022 [63]. Our study lacks information on behaviors that might affect the risk of SARS-CoV-2 infection such as wearing masks, sharing objects, utensil, toilets, and rooms with the index case [64,65,66,67]. However, such behaviors are not expected to be related to vaccination status, therefore their impact on the results is likely limited.

Our study has several strengths, including the prospective study design with a pre-defined sampling scheme of nasal follow-up swabs, with a high coverage of repeated sampling (swabs 1 and 2). Since we collected follow-up swabs on multiple time points after the identification of the index cases, we maximized the chances of capturing most COVID-19 infections that occurred in these households. Indeed, in our study, we found evidence of infection in 57.3% of household contacts during the Omicron period and 50.0% during the Delta period, compared with 15.0% and 10.8%, respectively, in a recent study from England that utilized routine data from national surveillance systems [41]. Additionally, we collected comprehensive information on COVID-19 vaccination status, including the number of doses received, vaccine brand, and timing of vaccination; and this information was validated against national vaccination records. Lastly, we captured COVID-19 waves caused by various variants, including the Alpha, Delta, and Omicron, which broadens the generalizability of our findings.

In conclusion, COVID-19 vaccination provides significant direct protection against SARS-CoV-2 infection in household contacts of COVID-19 index cases; however, this effect was blunted following the emergence of the Delta variant and more so during the Omicron wave, necessitating the administration of booster doses, even in setting with high vaccine uptake. COVID-19 vaccination in the index cases was independently related to reduced risk of infection in the household contacts, which might suggest indirect protection. Moreover, COVID-19 vaccination might reduce the shedding of the virus.

## Figures and Tables

**Table 1:** Description of the participants.

Characteristic	Index Cases	Household Contacts
Total	450	614
Sex		
Male	176 (39.1%)	321 (52.3%)
Female	274 (60.9%)	293 (47.7%)
Age in years		
Median, IQR	50 (17)	42 (35)
Min–Max	21–87	1–87
Age groups		
1–4	0 (0.0%)	16 (2.6%)
5–15	0 (0.0%)	91 (14.8%)
16–19	0 (0.0%)	34 (5.5%)
20–29	28 (6.2%)	71 (11.6%)
30–39	69 (15.3%)	71 (11.6%)
40–49	119 (26.4%)	104 (17.0%)
50–59	147 (32.7%)	133 (21.7%)
60–87	87 (19.3%)	93 (15.2%)
Ethnicity		
White	419 (93.1%)	558 (91.0%)
Asian (Indian/Pakistani)	11 (2.4%)	18 (2.9%)
Other	20 (4.4%)	37 (6.0%)
COVID-19 vaccination		
Not vaccinated	69 (15.3%)	211 (34.4%)
1 dose	85 (18.9%)	95 (15.5%)
2 doses	202 (44.9%)	227 (37.0%)
3 doses	94 (20.9%)	80 (13.0%)
4 doses	0 (0.0%)	1 (0.1%)

**Table 2:** COVID-19 vaccination status by demographic factors among index cases and household contacts aged 20 years or older.

	Index Cases N = 450				Household Contacts N = 472			
	Not Vaccinated	1 Dose	2 Doses	3 Doses	Not Vaccinated	1 Dose	2 Doses	3 Doses
Total	69	85	202	94	93	82	217	80
Age								
20–29	14 (20.3%)	5 (5.9%)	9 (4.5%)	0 (0.0%)	28 (30.1%)	19 (23.2%)	22 (10.2%)	2 (2.5%)
30–39	15 (21.7%)	17 (20.0%)	27 (13.4%)	10 (10.6%)	29 (31.2%)	6 (7.3%)	23 (10.6%)	13 (16.3%)
40–49	15 (21.7%)	21 (24.7%)	58 (28.7%)	25 (26.5%)	16 (17.2%)	14 (17.1%)	60 (27.8%)	14 (17.5%)
50–59	19 (27.5%)	22 (25.9%)	75 (37.1%)	31 (33.0%)	12 (12.9%)	29 (35.4%)	62 (28.7%)	30 (37.5%)
60–87	6 (8.7%)	20 (23.5%)	33 (16.3%)	28 (29.8%)	8 (8.6%)	14 (17.1%)	50 (23.0%)	21 (26.3%)
Sex								
Male	26 (37.7%)	34 (40.0%)	80 (39.6%)	36 (38.3%)	60 (64.5%)	39 (47.6%)	110 (50.7%)	45 (56.3%)
Female	43 (62.3%)	51 (60.0%)	122 (60.4%)	58 (61.7%)	33 (35.5%)	43 (52.4%)	107 (49.3%)	35 (43.8%)
Ethnicity								
White	64 (92.8%)	77 (90.6%)	191 (94.6%)	87 (92.6%)	84 (90.3%)	73 (90.1%)	200 (92.2%)	77 (96.2%)
Other	5 (7.2%)	8 (9.4%)	11 (5.4%)	7 (7.4%)	9 (9.7%)	8 (9.9%)	17 (7.8%)	3 (3.8%)
Household size								
2–3 individuals	40 (58.0%)	53 (62.4%)	119 (59.2%)	64 (68.1%)	41 (44.1%)	57 (69.5%)	132 (61.1%)	55 (68.8%)
4–8 individuals	29 (42.0%)	32 (37.6%)	82 (40.8%)	30 (31.9%)	52 (55.9%)	25 (30.5%)	84 (38.9%)	25 (31.2%)
Variant period								
Alpha (weeks 6–18, 2021)	32 (46.4%)	74 (87.1%)	11 (5.4%)	0 (0.0%)	57 (61.3%)	61 (77.4%)	9 (4.1%)	0 (0.0%)
Delta (weeks 19–48, 2021)	29 (42.0%)	11 (12.9%)	174 (86.1%)	45 (47.9%)	29 (31.2%)	21 (25.5%)	188 (86.6%)	32 (40.0%)
Omicron (weeks 49–52, 2021 & 1–6, 2022)	8 (11.6%)	0 (0.0%)	17 (8.4%)	49 (52.1%)	7 (7.5%)	0 (0.0%)	20 (9.2%)	48 (60.0%)

**Table 3:** SARS-CoV-2 RT-PCR positivity in at least one follow-up nasal swab among household contacts aged 20 years or older (N = 472) by demographics and COVID-19 vaccination status.

	Overall	Any Positive Nasal PCR	Unadjusted RR (95% CI) ^a^	*p* Value ^a^	Adjusted RR (95% CI) ^a^	*p* Value ^a^
	N = 472	238 (50.4%)				
Age group				0.016		0.006
20–29	71	27 (38.0%)	1.00		1.00	
30–39	71	44 (62.0%)	1.72 (1.19–2.49)	0.004	1.78 (1.26–2.54)	0.001
40–49	104	58 (55.8%)	1.57 (1.09–2.26)	0.014	1.75 (1.24–2.49)	0.001
50–59	133	59 (44.4%)	1.23 (0.85–1.90)	0.266	1.36 (0.95–1.95)	0.091
60–87	93	50 (53.8%)	1.54 (1.06–2.23)	0.023	1.57 (1.08–2.27)	0.018
Sex				0.872		0.945
Male	254	128 (50.4%)	1.00		1.00	
Female	218	110 (50.5%)	1.01 (0.84–1.22)		1.00 (0.84–1.20)	
Household size				0.298		0.046
2–3 individuals	285	149 (52.3%)	1.00		1.00	
4–8 individuals	186	89 (47.3%)	0.90 (0.75–1.09)		0.81 (0.66–0.99)	
Variant Period				0.344		<0.001
Alpha (weeks 6–18, 2021)	127	60 (47.2%)	1.00		1.00	
Delta (weeks 19–48, 2021)	270	135 (50.0%)	1.03 (0.83–1.29)	0.736	1.36 (1.04–1.79)	0.025
Omicron (weeks 49–52, 2021 & 1–6, 2022)	75	43 (57.3%)	1.20 (0.92–1.59)	0.173	2.01 (1.41–2.86)	<0.001
COVID-19 vaccination of the contact				0.104		<0.001
Not vaccinated	93	55 (59.1%)	1.00		1.00	
1 dose	82	38 (46.3%)	0.78 (0.58–1.04)	0.097	0.80 (0.59–1.09)	0.171
2 doses	217	112 (51.6%)	0.86 (0.70–1.07)	0.196	0.66 (0.50–0.86)	0.002
3 doses	80	33 (41.3%)	0.69 (0.50–0.95)	0.023	0.40 (0.28–0.59)	<0.001
The contact’s COVID-19 vaccination x Brand				0.033		<0.001
Not vaccinated	93	55 (59.1%)	1.00		1.00	
1 dose ChAdOx1-S	49	27 (55.1%)	0.93 (0.68–1.26)	0.648	1.00 (0.72–1.40)	0.983
1 dose mRNA vaccine	33	11 (33.3%)	0.56 (0.33–0.94)	0.028	0.58 (0.34–0.97)	0.040
2 doses ChAdOx1-S	137	79 (57.7%)	0.97 (0.77–1.21)	0.787	0.75 (0.57–1.00)	0.052
2 doses mRNA vaccine	80	33 (41.3%)	0.69 (0.51–0.95)	0.023	0.55 (0.39–0.78)	<0.001
3 doses mRNA vaccine	45	17 (37.8%)	0.63 (0.42–0.96)	0.033	0.37 (0.23–0.59)	<0.001
3 doses (2 ChAdOx1-S booster mRNA)	33	15 (45.5%)	0.76 (0.51–1.15)	0.209	0.46 (0.29–0.72)	<0.001
The contact’s COVID-19 vaccination x time				<0.001		<0.001
Not vaccinated, vaccinated 1 dose ≤ 21 days	105	65 (61.9%)	1.00		1.00	
Vaccinated 1 dose > 21 days or 2 doses ≤ 7 days	72	31 (43.1%)	0.69 (0.51–0.94)	0.020	0.72 (0.52–0.98)	0.042
Vaccinated 2 doses 8–90 days	74	28 (38.7%)	0.61 (0.44–0.84)	0.003	0.50 (0.35–0.72)	<0.001
Vaccinated 2 doses > 90 days or 3 doses ≤ 7 days	144	85 (59.0%)	0.95 (0.77–1.16)	0.645	0.69 (0.53–0.90)	0.007
Vaccinated 3 doses 8–151 days	76	29 (38.2%)	0.61 (0.44–0.85)	0.003	0.34 (0.23–0.50)	<0.001

^a^ Generalized Estimating Equations using negative binomial models, yielding relative risk (RR), 95% confidence intervals (CI) and *p* values. The variables included in the models were: vaccination status of the household contact, age, sex, household size, and period, as indicated in the table. The results of the covariates relate to the model in which COVID-19 vaccination status was analyzed as ‘the number of doses’. The results for the covariates in the remaining models related to ‘vaccine brand’ and ‘number of doses by time elapsed since vaccination’ were similar and thus not presented. ChAdOx1-S: the adenovirus vector vaccine (Oxford-AstraZeneca) COVID-19 vaccine, mRNA vaccine: either mRNA-1273 (Moderna, Inc.) or BNT162b2 (Pfizer-BioNTech) vaccines.

**Table 4:** The association between COVID-19 vaccination status of the index cases and household contacts with SARS-CoV-2 infection among household contacts aged 20 years or older.

	Overall					Delta Period				
	No. Positive PCR/Total Contacts (% Positive)	Unadjusted RR (95% CI) ^a^	*p* Value	Adjusted RR (95% CI) ^a,b^	*p* Value	No. Positive PCR/Total Contacts (% Positive)	Unadjusted RR (95% CI) ^a^	*p* Value	Adjusted RR (95% CI) ^a,b,c^	*p* Value
COVID-19 vaccination of their index case			0.077		0.015			0.021		0.023
Not vaccinated	40/71 (56.3%)	1.00		1.00		21/33 (63.6%)	1.00		1.00	
1 dose	46/92 (50.0%)	0.88 (0.66–1.18)	0.419	1.18 (0.79–1.76)	0.397	5/10 (50.0%)	0.78 (0.40–1.53)	0.481	0.52 (0.26–1.04)	0.068
2 doses	116/214 (54.2%)	0.95 (0.75–1.21)	0.727	1.13 (0.81–1.59)	0.445	97/182 (53.3%)	0.83 (0.62–1.12)	0.233	0.90 (0.59–1.36)	0.618
3 doses	36/95 (37.6%)	0.67 (0.48–0.93)	0.018	0.63 (0.40–0.98)	0.045	12/45 (26.7%)	0.41 (0.24–0.72)	0.002	0.45 (0.23–0.89)	0.021
COVID-19 vaccination of the household contact			0.104		0.008			0.004		<0.001
Not vaccinated	55/93 (59.1%)	1.00		1.00		17/29 (58.6%)	1.00		1.00	
1 dose	38/82 (46.3%)	0.78 (0.58–1.04)	0.097	0.81 (0.58–1.11)	0.146	15/21 (71.4%)	1.21 (0.81–1.83)	0.343	1.46 (0.81–2.62)	0.200
2 doses	112/217 (51.6%)	0.86 (0.70–1.07)	0.196	0.61 (0.42–0.88)	0.009	97/187 (51.9%)	0.88 (0.62–1.23)	0.458	0.64 (0.37–1.12)	0.121
3 doses	33/80 (41.3%)	0.69 (0.50–0.95)	0.023	0.42 (0.25–0.69)	<0.001	6/33 (18.2%)	0.31 (0.14–0.68)	0.003	0.28 (0.11–0.73)	0.009

^a^ Generalized Estimating Equations using negative binomial models yielding relative risk (RR), 95% confidence intervals (CI) and *p* values. ^b^ A multivariable model included age, sex, household size, vaccination status of the index case, vaccination status of the household contact, and calendar month-year as independent variables. ^c^ The analysis was restricted to individuals recruited during the Delta wave (calendar weeks 18–48 2021). The overall analysis was based on 450 cases and 472 household contacts; the analysis of the Delta period was based on 259 index cases and 270 household contacts.

**Table 5:** The association of COVID-19 vaccination and Ct values of the Orf gene of SARS-CoV-2 positive participants on their first positive swab–pooled index cases and household contacts analysis.

	Number	Ct Value ≤ 25.02 (Median)	Ct Value > 25.02 (Median)	Adjusted RR (95% CI) ^a^	*p* Value
Not vaccinated	114	67 (58.8%)	47 (41.2%)	1.00	
1 dose	105	62 (59.0%)	43 (41.0%)	0.98 (0.89–1.08)	0.809
2 doses	289	150 (51.9%)	139 (48.1%)	1.05 (0.96–1.14)	0.263
3 doses	104	41 (39.4%)	63 (60.6%)	1.12 (1.01–1.25)	0.027

^a^ Generalized Estimating Equations using negative binomial models yielding relative risk (RR), 95% confidence intervals (CI) and *p* values. The model included Ct values as the dependent variable, comparing high Ct values (above the median) to lower Ct values. The independent variable was vaccination status, the model adjusted for index case/household contact status and variant periods.

## Data Availability

The raw study data are protected and are not freely available due to data privacy laws. This work is carried out under Regulation 3 of The Health Service (Control of Patient Information) (Secretary of State for Health, 2002) (3) using patient identification information.

## Supplementary Materials

The following supporting information can be downloaded at: https://www.mdpi.com/article/10.3390/vaccines12020113/s1, Supplementary methods, Supplementary Figure S1: Enrollment of index cases and contacts by month/year: COVID-19 household study; Supplementary Table S1: Characteristics of individuals who were included and those who were excluded from the study; Supplementary Table S2: Symptoms among the index cases and SARS-CoV-2 RT-PCR positive household contacts aged 20 years or older; Supplementary Table S3: COVID-19 vaccination status by demographic factors among all household contacts; Supplementary Table S4: COVID-19 vaccination status among the index cases and their household contacts aged 20 years or older; Supplementary Table S5A: SARS-CoV-2 RT-PCR positivity in at least one follow-up nasal swab among the index cases by demographics and COVID-19 vaccination status; Supplementary Table S5B: SARS-CoV-2 positivity in the follow-up nasal RT-PCR swabs among the index cases by demographics and COVD-19 vaccination status; Supplementary Table S5C: Number of index cases with missing follow-up nasal RT-PCR swabs; Supplementary Table S6A: SARS-CoV-2 PCR positivity in the follow-up nasal swabs among the household contacts aged 20 years or older by demographics and COVID-19 vaccination status; Supplementary Table S6B: Number of household contacts aged 20 years or older with missing follow-up nasal PCR swabs; Supplementary Table S7: COVID-19 (symptomatic SARS-CoV-2 infection) among household contacts aged 20 years or older by demographics and COVID-19 vaccination status—a sensitivity analysis; Supplementary Table S8: The associations of COVID-19 vaccination with overall SARS-CoV-2 infection and COVID-19 among household contacts aged 20 years or older-sensitivity analyses; Supplementary Table S9: COVID-19 vaccination status of household contacts aged 20 years or older by variant period; Supplementary Table S10. The associations of COVID-19 vaccination with overall SARS-CoV-2 infection among household contacts aged 20 years or older during the Alpha variant period (N = 126)—a sensitivity analysis; Supplementary Table S11: The associations of COVID-19 vaccination with overall SARS-CoV-2 infection among household contacts aged 20 years or older during the Delta variant period (N = 270); Supplementary Table S12. The associations of COVID-19 vaccination with overall SARS-CoV-2 infection among household contacts aged 20 years or older during the Omicron variant period (N = 75); Supplementary Table S13: The correlation between the number of COVID-19 vaccination received by the index cases and their household contacts; Supplementary Table S14: The association of COVID-19 vaccination and Ct values of the E gene of SARS-CoV-2 positive participants on their first positive swab–a pooled analysis of index cases and household contacts.
