# Supplementary material for: Association between COVID-19 Vaccination and SARS-CoV-2 Infection among Household Contacts of Infected Individuals: A Prospective Household Study in England
Source: Vaccines (Basel). 2024 Jan 23;12(2):113. doi: 10.3390/vaccines12020113 (PMC10892628; doi:10.3390/vaccines12020113)
Supplement: Supplementary file 1 [file vaccines-12-00113-s001.zip › vaccines-2781309-supplementary.pdf]

Supplementary Materials

Sensitivity analysis stratified by variant period

In the stratified analysis by study period, we categorized the vaccination status of the household contacts in a manner that reflects the vaccination policy during each period. During the Alpha wave, most individuals received one dose of a COVID-19 vaccine and only a few received two doses. During the Delta wave, booster vaccination was introduced, therefore vaccination status was classified similarly to the main analysis. During the Omicron wave, most individuals were vaccinated with two doses (mostly >90 days), or three doses; accordingly, they were classified as vaccinated with three doses ≥ eight days (i.e. protected), or else (including unvaccinated, vaccinated with two doses (regardless of time elapsed since vaccination) or three doses ≤seven days).

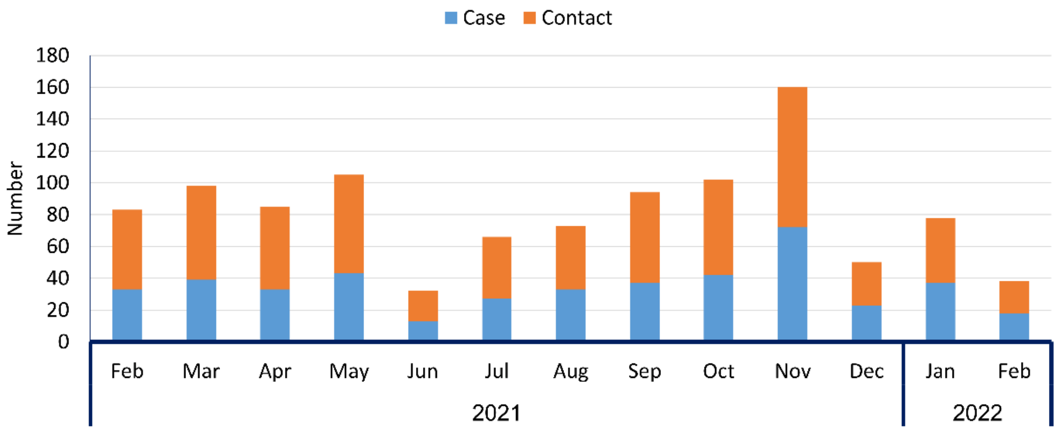

The enrollment date was determined based on the date of the interview

Supplementary Figure S1. Enrollment of index cases and contacts by month/year: COVID-19 household study.

Supplementary Table S1. Characteristics of individuals who were included and those who were excluded from the study.

Characteristic	Included in the study	Excluded from the study
Total	1064	154
Status		
Index case	450 (42.3%)	63 (40.9%)
Household contact	614 (57.7%)	92 (59.1%)
Sex		
Male	497 (46.7%)	69 (44.8%)
Female	567 (53.3%)	85 (55.2%)
Age groups, years		
1-4	16 (1.5%)	5 (3.2%)
5-15	91 (8.6%)	21 (13.6%)
16-19	34 (3.2%)	8 (5.2%)
20-29	99 (9.3%)	17 (11.0%)
30-39	140 (13.2%)	26 (16.9%)
40-49	223 (21.0%)	28 (18.2%)
50-59	280 (26.3%)	23 (14.9%)
60-87	180 (16.9%)	26 (16.9%)
Ethnicity		
White	977 (91.9%)	140 (90.9%)
Other	88 (8.1%)	14 (9.1%)
Household size		
2-3 individuals	585 (55.1%)	82 (53.2%)
4-8 individuals	477 (44.9%)	72 (46.8%)
Variant/period		
Alpha (week 6-18, 2021)	299 (28.1%)	52 (33.8%)
Delta (weeks 19-48, 2021)	607 (57.0%)	90 (58.4%)
Omicron (weeks 49-52 2021/weeks 1-6, 2022)	158 (14.8%)	12 (7.8%)
COVID-19 vaccination (age ≥20 years) (n=1042)	922	120
Not vaccinated	162 (17.6%)	32 (26.7%)
1 dose	167 (18.1%)	17 (14.2%)
2 doses	419 (45.4%)	67 (55.8%)
3 doses	174 (18.9%)	4 (3.3%)

Supplementary Table S2. Symptoms among the index cases and SARS-CoV-2 RT-PCR positive household contacts aged 20 years or older.

	**Index cases**	**Positive household contacts**
Total	450	238
Fever	241 (53.6%)	120 (50.4%)
Cough	334 (74.2%)	169 (71.0%)
Loss of smell/taste	227 (50.4%)	118 (49.6%)
Other symptoms	420 (93.3%)	227 (95.4%)
Fever, cough, or loss of smell/taste	390 (86.7%)	200 (84.0%)
Any symptom	427 (94.9%)	229 (96.2%)
Number of symptoms		
0	23 (5.1%)	9 (3.8%)
1	44 (9.8%)	31 (13.0%)
2	110 (24.4%)	57 (23.9%)
3	134 (29.8%)	75 (31.5%)
≥4	139 (30.9%)	66 (27.7%)

Supplementary Table S3. COVID-19 vaccination status by demographic factors among all household contacts.

	Household contacts, N=614			
	**Not vaccinated**	**1 dose**	2 doses	3 doses [a]
Total	211	95	227	81
Age groups, years [b]				
1-4	16 (7.6%)	0 (0.0%)	0 (0.0%)	0 (0.0%)
5-15	85 (40.3%)	4 (4.2%)	2 (0.9%)	0 (0.0%)
16-19	17 (8.1%)	9 (9.5%)	8 (3.5%)	0 (0.0%)
20-29	28 (13.3%)	19 (20.0%)	22 (9.7%)	2 (2.5%)
30-39	29 (13.7%)	6 (6.3%)	23 (10.2%)	13 (16.0%)
40-49	16 (7.6%)	14 (14.7%)	60 (26.5%)	14 (17.3%)
50-59	12 (5.7%)	29 (30.5%)	62 (27.4%)	30 (37.0%)
60-87	8 (3.8%)	14 (14.7%)	49 (21.7%)	22 (27.2%)
Sex				
Male	118 (55.9%)	45 (47.4%)	112 (49.3%)	46 (56.8%)
Female	93 (44.1%)	50 (52.6%)	115 (50.7%)	35 (43.2%)
Ethnicity				
White	186 (88.2%)	85 (90.4%)	209 (92.1%)	78 (96.3%)
Other	25 (11.8%)	9 (9.6%)	18 (7.9%)	3 (3.7%)
Household size				
2-3 individuals	59 (28.0%)	57 (60.0%)	137 (60.6%)	56 (69.1%)
4-8 individuals	152 (72.0%)	38 (40.0%)	89 (39.4%)	25 (30.9%)
Period				
Alpha (week 6-18, 2021)	110 (52.1%)	63 (66.3%)	9 (4.0%)	0 (0.0%)
Delta (weeks 19-48, 2021)	91 (43.1%)	31 (32.6%)	194 (85.5%)	32 (39.5%)
Omicron (weeks 49-52 2021/weeks 1-6, 2022)	10 (4.7%)	1 (1.1%)	24 (10.5%)	49 (60.5%)

[a] One person who received 4 doses was included in the 3 doses group. [b] Information on age was missing for 1 person.

Supplementary Table S4. COVID-19 vaccination status among the index cases and their household contacts aged 20 years or older.

	**Index cases**	**Household contacts**
Total	450	472
Not vaccinated	69 (15.3%)	93 (19.7%)
1 dose	85 (18.9%)	82 (17.4%)
2 doses	202 (44.9%)	217 (46.0%)
3 doses	94 (20.9%)	80 (16.9%)
1 dose ChAdOx1-S	42 (9.3%)	49 (10.4%)
1 dose mRNA vaccine	43 (9.6%)	33 (7.0%)
2 doses ChAdOx1-S	141 (31.3%)	137 (29.0%)
2 doses mRNA vaccine	61 (13.6%)	80 (16.9%)
3 doses mRNA vaccine	49 (10.9%)	45 (9.5%)
3 doses (2 ChAdOx1-S booster mRNA)	45 (10.0%)	33 (7.0%)
Other	0 (0.0%)	2 (0.4%)
1 dose≤21 days	13 (2.9%)	12 (2.5%)
1 dose>21 days	72 (16.0%)	69 (14.6%)
2 doses≤7 days	11 (2.4%)	3 (0.6%)
2 doses 8-90 days	73 (16.3%)	74 (15.7%)
2 doses>90 days	118 (26.3%)	140 (29.7%)
3 doses≤7 days	15 (3.3%)	4 (0.8%)
3 doses 8-151 days	78 (17.4%)	76 (16.1%)

ChAdOx1-S: the adenovirus vector vaccine (Oxford-AstraZeneca) COVID-19 vaccine, mRNA vaccine: either mRNA-1273 (Moderna, Inc.) or BNT162b2 (Pfizer-BioNTech) vaccines.

Supplementary Table S5. A: SARS-CoV-2 RT-PCR positivity in at least one follow-up nasal swab among the index cases by demographics and COVID-19 vaccination status.

	Overall	Any positive nasal PCR	Unadjusted RR (95% CI)[a]	P value	Adjusted RR (95% CI)[a]	P value
	450	398 (88.4%)				
**Age group**				0.735		0.732
20-29	28	25 (89.3%)	1.00		1.00	
30-39	69	64 (92.8%)	1.01 (0.90-1.14)	0.793	1.03 (0.91-1.18)	0.571
40-49	119	104 (87.4%)	0.98 (0.86-1.10)	0.768	1.01 (0.88-1.15)	0.846
50-59	147	127 (86.4%)	0.95 (0.84-1.08)	0.493	0.97 (0.86-1.11)	0.735
60-87	87	78 (89.7%)	0.98 (0.87-1.11)	0.835	1.01 (0.88-1.16)	0.840
**Sex**				0.045		0.064
Male	176	164 (93.2%)	1.00		1.00	
Female	274	234 (85.4%)	0.94 (0.89-0.99)		0.94 (0.89-1.00)	
**Household size**				0.140		0.979
2-3 individuals	276	243 (88.0%)	1.00		1.00	
4-8 individuals	173	154 (89.0%)	1.01 (0.95-1.07)		0.99 (0.92-1.07)	
**Period**				0.769		0.295
Alpha (weeks 6-18, 2021)	117	100 (85.5%)	1.00		1.00	
Delta (weeks 19-48, 2021)	259	231 (89.2%)	1.02 (0.95-1.10)	0.470	1.11 (0.96-1.29)	0.130
Omicron (weeks 49-52 2021 & 1-6, 2022)	74	67 (90.5%)	1.02 (0.92-1.13)	0.646	1.03 (0.93-1.15)	0.475
**COVID-19 vaccination of the case**				0.041		0.060
Not vaccinated	69	64 (92.8%)	1.00		1.00	
1 dose	85	74 (87.1%)	0.96 (0.86-1.06)	0.424	0.97 (0.86-1.10)	0.714
2 doses	202	184 (91.1%)	1.02 (0.94-1.10)	0.569	1.00 (0.92-1.10)	0.850
3 doses	94	76 (80.9%)	0.89 (0.79-1.00)	0.052	**0.84 (0.73-0.98)**	**0.027**
**COVID-19 vaccination x Brand (index cases)**				0.114		0.083
Not vaccinated	69	64 (92.8%)	1.00		1.00	
1 dose ChAdOx1-S	42	40 (95.2%)	1.02 (0.93-1.12)	0.583	1.04 (0.93-1.17)	0.405
1 dose mRNA vaccine	43	34 (79.1%)	0.88 (0.76-1.04)	0.144	0.90 (0.76-1.07)	0.257
2 doses ChAdOx1-S	141	133 (94.3%)	1.02 (0.94-1.10)	0.545	1.01 (0.92-1.11)	0.716
2 doses mRNA vaccine	61	51 (83.6%)	1.01 (0.92-1.11)	0.742	1.00 (0.90-1.12)	0.905
3 doses mRNA vaccine	49	40 (81.6%)	0.91 (0.79-1.04)	0.194	0.87 (0.74-1.03)	0.115
3 doses (2 ChAdOx1-S, booster mRNA)	45	36 (80.0%)	0.86 (0.73-1.02)	0.091	**0.82 (0.68-0.99)**	**0.047**
**COVID-19 vaccination x time (index cases)**	N=449			0.508		0.082
Not vaccinated, vaccinated 1 dose ≤21 days	82	76 (92.7%)	1.00		1.00	
Vaccinated 1 dose>21 days or 2 doses≤7 days	83	72 (86.7%)	0.93 (0.84-1.04)	0.211	0.96 (0.86-1.07)	0.480
Vaccinated 2 doses 8-90 days	73	64 (87.7%)	0.94 (0.85-1.05)	0.301	0.90 (0.79-1.03)	0.139
Vaccinated 2 doses>90 days or 3 doses≤7 days	133	119 (89.5%)	0.96 (0.88-1.05)	0.412	0.90 (0.81-1.00)	0.072
Vaccinated 3 doses 8-151 days	78	66 (84.6%)	0.91 (0.81-1.02)	0.113	**0.83 (0.71-0.96)**	**0.017**

[a] Generalized Estimating Equations using negative binomial models yielding relative risk (RR), 95% confidence intervals (CI) and P values. The variables that were included in the models were: vaccination status of the case, age, sex, household size, and period, as indicated in the table. The results of the covariates relate to the model in which COVID-19 vaccination status was introduced as the number of doses. The results for the covariates in the remaining models related to 'vaccine brand' and number of doses by time elapsed since vaccination' were similar and thus not presented. ChAdOx1-S: the adenovirus vector vaccine (Oxford-AstraZeneca) COVID-19 vaccine, mRNA vaccine: either mRNA-1273 (Moderna, Inc.) or BNT162b2 (Pfizer-BioNTech) vaccines.

Supplementary Table S5. B: SARS-CoV-2 positivity in the follow-up nasal RT-PCR swabs among the index cases by demographics and COVD-19 vaccination status[a].

	Overall	Nasal PCR swab 1	P value	Nasal PCR swab 2 [a]	P value	Nasal PCR swab 3 [a]	P value	All PCRs positives [a]	P value	All PCRs negatives [a]	P value
		N=450		N=439		N=420		N=420		N=420	
**Overall**	450	391 (86.9%)		327 (74.5%)		181 (43.1%)		174 (41.4%)		44 (10.5%)	
**Age group**			0.958		0.667		0.012		0.017		0.365
20-29	28	24 (85.7%)		21 (84.0%)		10 (52.6%)		10 (52.6%)		1 (5.3%)	
30-39	69	62 (89.9%)		48 (75.0%)		31 (51.7%)		30 (50.0%)		3 (5.0%)	
40-49	119	103 (86.6%)		85 (72.0%)		38 (32.8%)		37 (31.9%)		14 (12.1%)	
50-59	147	127 (86.4%)		106 (72.6%)		57 (39.9%)		54 (37.8%)		19 (13.3%)	
60-87	87	75 (86.2%)		67 (77.9%)		45 (54.9%)		43 (52.4%)		7 (8.5%)	
**Sex**			0.146		0.227		0.016		0.031		0.007
Male	176	158 (89.8%)		132 (77.6%)		83 (50.3%)		79 (47.9%)		9 (5.5%)	
Female	274	233 (85.0%)		195 (72.5%)		98 (38.4%)		95 (37.3%)		35 (13.7%)	
**Household size**			0.619		0.423		0.064		0.064		0.949
2-3 individuals	276	239 (86.2%)		206 (75.7%)		121 (46.7%)		121 (46.7%)		27 (10.4%)	
4-8 individuals	173	152 (87.9%)		120 (72.3%)		60 (37.5%)		60 (37.5%)		17 (10.6%)	
**Period**			0.269		0.353		0.419		0.368		0.280
Alpha (week 6-18, 2021)	117	97 (82.9%)		90 (78.9%)		50 (45.0%)		50 (45.0%)		15 (13.5%)	
Delta (weeks 19-48, 2021)	259	227 (87.6%)		183 (72.0%)		107 (42.4%)		101 (41.7%)		25 (10.3%)	
Omicron (weeks 49-52 2021/weeks 1-6, 2022)	74	67 (90.5%)		54 (76.1%)		24 (35.8%)		23 (34.3%)		4 (6.0%)	
**COVID-19 vaccination**			0.046		0.007		0.070		0.033		0.063
Not vaccinated	69	63 (91.3%)		59 (88.1%)		35 (57.4%)		35 (57.4%)		4 (6.6%)	
1 dose	85	71 (83.5%)		65 (80.2%)		32 (41.0%)		32 (41.0%)		8 (10.3%)	
2 doses	202	182 (90.1%)		141 (71.2%)		82 (42.7%)		77 (40.1%)		16 (8.3%)	
3 doses	94	75 (79.8%)		62 (66.7%)		32 (36.0%)		30 (33.7%)		16 (18.0%)	
**COVID-19 vaccination x Brand**			0.004		0.011		0.198		0.046		0.022
Not vaccinated	69	63 (91.3%)		59 (88.1%)		35 (57.4%)		35 (57.4%)		4 (6.6%)	
1 dose ChAdOx1-S	42	38 (90.5%)		33 (82.5%)		17 (43.6%)		17 (43.6%)		2 (5.1%)	
1 dose mRNA vaccine	43	33 (76.7%)		32 (78.0%)		15 (38.5%)		15 (38.5%)		6 (15.4%)	
2 doses ChAdOx1-S	141	133 (94.3%)		106 (75.2%)		62 (45.3%)		61 (44.5%)		7 (5.1%)	
2 doses mRNA vaccine	61	49 (80.3%)		35 (61.4%)		20 (36.4%)		16 (29.1%)		9 (16.4%)	
3 doses mRNA vaccine	49	40 (81.6%)		31 (64.6%)		17 (37.0%)		16 (34.8%)		8 (17.4%)	
3 doses (2 ChAdOx1-S booster mRNA)	45	35 (77.8%)		31 (68.9%)		15 (34.9%)		14 (32.6%)		8 (18.6%)	
**COVID-19 vaccination x time**	N=449[b]		0.004	N=438 [c]	0.003	N=419 [c]	0.062	N=419 [c]	0.039	N=419 [c]	0.028
Not vaccinated	69	63 (91.3%)		59 (88.1%)		35 (57.4%)		35 (57.4%)		4 (6.6%)	

1 dose≤21 days	13	12 (92.3%)	10 (83.3%)	6 (50.0%)	6 (50.0%)	1 (8.3%)
1 dose>21 days	72	59 (81.9%)	55 (79.9%)	26 (39.4%)	26 (39.4%)	7 (10.6%)
2 doses≤7 days	11	10 (90.9%)	6 (54.5%)	4 (36.4%)	3 (27.3%)	1 (9.1%)
2 doses 8-90 days	73	64 (87.7%)	45 (62.5%)	31 (44.9%)	29 (42.0%)	9 (13.0%)
2 doses>90 days	118	108 (91.5%)	90 (78.3%)	47 (42.0%)	45 (40.2%)	6 (5.4%)
3 doses≤7 days	15	8 (53.3%)	8 (53.3%)	1 (7.1%)	1 (7.1%)	5 (35.7%)
3 doses 8-151 days	78	66 (84.6%)	53 (68.8%)	31 (41.9%)	29 (39.2%)	11 (14.9%)

[a] The numbers in the different categories might not add to 450, since some did not provide second or third nasal follow-up swabs. [b] 1 case did not have information on the date vaccination. [c] Supplementary table 4B provides information on the number of cases with missing data, according to the different variables/categories. P values were obtained using the chi square test. ChAdOx1-S: the adenovirus vector vaccine (Oxford-AstraZeneca) COVID-19 vaccine, mRNA vaccine: either mRNA-1273 (Moderna, Inc.) or BNT162b2 (Pfizer-BioNTech) vaccines.

Supplementary Table S5. C: Number of index cases with missing follow-up nasal RT-PCR swabs.

	Overall	Number with missing nasal PCR swab 2	Number with missing nasal PCR swab 3
**Total**	450	11	30
**Age group**			
20-29	28	3	9
30-39	69	5	9
40-49	119	1	3
50-59	147	1	4
60-87	87	1	5
**Sex**			
Male	176	6	11
Female	274	5	16
**Household size**			
2-3 individuals	276	4	17
4-8 individuals	173	7	13
**Period**			
Alpha (week 6-18, 2021)	117	3	6
Delta (weeks 19-48, 2021)	259	5	17
Omicron (weeks 49-52 2021/weeks 1-6, 2022)	74	3	7
**COVID-19 vaccination**			
Not vaccinated	69	2	8
1 dose	85	4	7
2 doses	202	4	10
3 doses	94	1	5
1 dose ChAdOx1-S	42	2	3
1 dose mRNA vaccine	43	2	4
2 doses ChAdOx1-S	141	0	4
2 doses mRNA vaccine	61	4	6
3 doses mRNA vaccine	49	1	3
3 doses (2 ChAdOx1-S booster mRNA)	45	0	2
1 dose≤21 days	13	1	1
1 dose>21 days	72	3	6
2 doses≤7 days	11	0	0
2 doses 8-90 days	73	1	4
2 doses>90 days	118	3	6
3 doses≤7 days	15	0	1
3 doses 8-151 days	78	1	4

ChAdOx1-S: the adenovirus vector vaccine (Oxford-AstraZeneca) COVID-19 vaccine, mRNA vaccine: either mRNA-1273 (Moderna, Inc.) or BNT162b2 (Pfizer-BioNTech) vaccines.

Supplementary Table S6. A: SARS-CoV-2 PCR positivity in the follow-up nasal swabs among the household contacts aged 20 years or older by demographics and COVID-19 vaccination status [a.]

	Overall	Nasal PCR swab 1	P value	Nasal PCR swab 2 [a]	P value	Nasal PCR swab 3 [a]	P value	All nasal PCRs swabs positive [a]	P value	All nasal PCRs swabs negative [a]	P value
**Overall number of swabs at each time point**	N=472	N=472		N=458		N=430		N=430		N=430	
**Age group**			0.010		0.007		0.006		0.005		0.002
20-29	71	21 (29.6%)		21 (31.3%)		10 (17.2%)		6 (10.3%)		38 (65.5%)	
30-39	71	39 (54.5%)		37 (56.1%)		27 (42.2%)		23 (35.9%)		25 (39.1%)	
40-49	104	55 (52.9%)		47 (46.1%)		33 (34.5%)		30 (31.6%)		41 (43.2%)	
50-59	133	54 (40.6%)		46 (34.8%)		32 (23.6%)		26 (20.5%)		70 (55.1%)	
60-87	93	48 (51.6%)		45 (49.5%)		32 (37.2%)		30 (34.9%)		38 (44.2%)	
**Sex**			0.972		0.452		0.924		0.996		0.671
Male	254	117 (46.1%)		102 (41.3%)		71 (30.7%)		62 (26.8%)		115 (49.8%)	
Female	218	100 (45.9%)		94 (44.5%)		61 (30.7%)		53 (26.6%)		97 (48.7%)	
**Household size**			0.251		0.392		0.997		0.516		0.106
2-3 individuals	285	137 (48.1%		124 (44.3%)		80 (30.4%)		73 (27.8%)		122 (48.4%)	
4-8 individuals	186	79 (42.5%)		71 (40.1%)		51 (30.7%)		41 (24.7%)		90 (54.2%)	
**Period**			0.135		0.818		0.572		0.990		0.401
Alpha (weeks 6-18, 2021)	127	50 (39.4%)		48 (40.0%)		39 (34.2%)		30 (26.3%)		59 (51.8%)	
Delta (weeks 19-48, 2021)	270	126 (46.7%)		116 (43.8%)		72 (29.3%)		66 (26.8%)		123 (50.0%)	
Omicron (weeks 49-52 2021/ weeks 1-6, 2022)	75	41 (54.7%)		32 (43.8%)		21 (30.0%)		19 (27.1%)		30 (42.9%)	
**COVID-19 vaccination**			0.511		0.050		0.258		0.531		0.291
Not vaccinated	93	47 (50.5%)		45 (51.1%)		29 (36.3%)		24 (30.0%)		34 (42.5%)	
1 dose	82	34 (41.5%)		29 (36.7%)		22 (30.6%)		19 (26.4%)		37 (51.4%)	
2 doses	217	103 (47.5%)		97 (46.0%)		65 (32.0%)		57 (28.1%)		98 (48.3%)	
3 doses	80	33 (41.3%)		25 (31.6%)		16 (21.3%)		15 (20.0%)		43 (57.3%)	
**COVID-19 vaccination x Brand**			0.099		0.034		0.023		0.057		0.017
Not vaccinated	93	47 (50.5%)		45 (51.1%)		29 (36.3%)		24 (30.0%)		34 (42.5%)	
1 dose ChAdOx1-S	49	25 (51.0%)		21 (43.8%)		18 (40.9%)		16 (36.4%)		19 (43.2%)	
1 dose mRNA vaccine	33	9 (27.3%)		8 (25.8%)		4 (14.3%)		3 (10.7%)		18 (64.3%)	
2 doses ChAdOx1-S	137	73 (53.3%)		69 (50.7%)		50 (38.5%)		44 (33.8%)		54 (41.5%)	
2 doses mRNA vaccine	80	30 (37.5%)		28 (36.8%)		15 (20.5%)		13 (17.8%)		44 (60.3%)	
3 doses mRNA vaccine	45	17 (37.8%)		12 (27.3%)		10 (23.3%)		9 (20.9%)		27 (62.8%)	
3 doses (2 ChAdOx1-S, booster mRNA)	33	15 (45.5%)		12 (36.4%)		6 (20.0%)		6 (20.0%)		15 (50.0%)	
**COVID-19 vaccination x time**			<0.001		0.009		0.051		0.043		0.004
Not vaccinated	93	47 (50.5%)		45 (51.1%)		29 (36.3%)		24 (30.0%)		34 (42.5%)	

1 dose≤21 days	12	9 (75.0%)	6 (50.0%)	6 (54.5%)	5 (45.5%)	2 (18.2%)
1 dose>21 days	69	25 (36.2%)	23 (34.8%)	16 (26.2%)	14 (23.0%)	35 (57.4%)
2 doses≤7 days	3	3 (100.0%)	2 (66.7%)	3 (33.3%)	3 (33.3%)	0 (0.0%)
2 doses 8-90 days	74	23 (31.1%)	25 (34.2%)	16 (22.5%)	11 (15.5%)	45 (63.4%)
2 doses>90 days	140	77 (55.0%)	70 (51.5%)	48 (37.2%)	45 (34.9%)	53 (41.1%)
3 doses≤7 days	4	4 (100.0%)	3 (75.0%)	2 (50.0%)	2 (50.0%)	0 (0.0%)
3 doses 8-151 days	76	29 (38.2%)	22 (29.3%)	14 (19.7%)	13 (18.3%)	43 (60.6%)

[a] The numbers in the different categories might not add to 472, since some did not provide second or third nasal follow-up swabs. Supplementary table 5B provides information on the number of index cases with missing data, according to the different variables/categories P values were obtained using bivariate Generalized Estimating Equations using negative binomial models yielding relative risk (RR), 95% confidence intervals (CI) and P values. ChAdOx1-S: the adenovirus vector vaccine (Oxford-AstraZeneca) COVID-19 vaccine, mRNA vaccine: either mRNA-1273 (Moderna, Inc.) or BNT162b2 (Pfizer-BioNTech) vaccines.

Supplementary Table S6. B: Number of household contacts aged 20 years or older with missing follow-up nasal PCR swabs.

	**Overall**	**Number with missing nasal PCR swab 2**	**Number with missing nasal PCR swab 3**
Total	N=472	14	42
Age group			
20-29	71	4	13
30-39	71	5	7
40-49	104	2	9
50-59	133	1	6
60-87	93	2	7
Sex			
Male	254	7	23
Female	218	7	19
Household size			
2-3 individuals	285	5	12
4-8 individuals	196	9	30
Period			
Alpha (weeks 6-18, 2021)	127	7	13
Delta (weeks 19-48, 2021)	270	5	24
Omicron (weeks 49-52 2021/ weeks 1-6, 2022)	75	2	5
COVID-19 vaccination			
Not vaccinated	93	5	13
1 dose	82	3	10
2 doses	217	5	14
3 doses	80	1	5
1 dose ChAdOx1-S	49	1	5
1 dose mRNA vaccine	33	2	5
2 doses ChAdOx1-S	137	1	7
2 doses mRNA vaccine	80	4	7
3 doses mRNA vaccine	45	1	2
3 doses (2 ChAdOx1-S booster mRNA)	33	0	3
1 dose≤21 days	12	0	1
1 dose>21 days	69	3	8
2 doses≤7 days	3	0	0
2 doses 8-90 days	74	1	3
2 doses>90 days	140	4	11
3 doses≤7 days	4	0	0
3 doses 8-151 days	76	1	5

ChAdOx1-S: the adenovirus vector vaccine (Oxford-AstraZeneca) COVID-19 vaccine, mRNA vaccine: either mRNA-1273 (Moderna, Inc.) or BNT162b2 (Pfizer-BioNTech) vaccines.

Supplementary Table S7: COVID-19 (symptomatic SARS-CoV-2 infection) among household contacts aged 20 years or older by demographics and COVID-19 vaccination status – a sensitivity analysis.

	Overall	COVID-19	Unadjusted RR (95% CI) [a]	P value	Adjusted RR (95% CI)[a]	P value
**Age group**				0.014		0.005
20-29	69	25 (36.2%)	1.00		1.00	
30-39	69	42 (60.9%)	1.79 (1.21-2.64)	0.004	1.86 (1.28-2.69)	0.001
40-49	102	56 (54.9%)	1.62 (1.10-2.38)	0.013	1.85 (1.28-2.66)	0.001
50-59	130	56 (43.1%)	1.27 (0.85-1.88)	0.238	1.42 (0.98-2.07)	0.063
60-87	93	50 (53.8%)	1.61 (1.09-2.38)	0.016	1.70 (1.16-2.50)	0.007
**Sex**				0.941		0.860
Male	249	123 (49.4%)	1.00		1.00	
Female	214	106 (49.5%)	1.00 (0.83-1.21)		1.01 (0.84-1.22)	
**Household size**				0.261		0.070
2-3 individuals	279	143 (51.3%)	1.00		1.00	
4-8 individuals	183	85 (46.4%)	0.89 (0.73-1.08)		0.82 (0.66-1.01)	
**Variant Period**				0.411		<0.001
Alpha (weeks 6-18, 2021)	123	56 (45.5%)	1.00		1.00	
Delta (weeks 19-48, 2021)	267	132 (49.4%)	1.06 (0.84-1.34)	0.613	1.45 (1.10-1.93)	0.008
Omicron (weeks 49-52 2021 & 1-6, 2022)	73	41 (56.2%)	1.21 (0.90-1.61)	0.193	2.16 (1.50-3.10)	<0.001
**COVID-19 vaccination of the contact**				0.087		<0.001
Not vaccinated	91	53 (58.2%)	1.00		1.00	
1 dose	81	37 (45.7%)	0.78 (0.58-1.08)	0.106	0.81 (0.59-1.11)	0.196
2 doses	214	109 (50.9%)	0.87 (0.70-1.08)	0.214	0.63 (0.48-0.82)	0.001
3 doses	77	30 (39.0%)	0.66 (0.48-0.93)	0.017	0.36 (0.24-0.53)	<0.001
**The household contacts' COVID-19 vaccination x Brand**				0.026		<0.001
Not vaccinated	91	53 (58.2%)	1.00		1.00	
1 dose ChAdOx1-S	48	26 (54.2%)	0.93 (0.68-1.27)	0.650	1.00 (0.71-1.41)	0.990
1 dose mRNA vaccine	33	11 (33.3%)	0.57 (0.34-0.95)	0.033	0.60 (0.35-1.00)	0.053
2 doses ChAdOx1-S	135	77 (57.0%)	0.97 (0.77-1.22)	0.820	0.72 (0.54-0.95)	0.025
2 doses mRNA vaccine	79	32 (40.5%)	0.69 (0.50-0.95)	0.026	0.53 (0.37-0.75)	<0.001
3 doses mRNA vaccine	42	14 (33.3%)	0.57 (0.36-0.90)	0.018	0.31 (0.19-0.50)	<0.001
3 doses (2 ChAdOx1-S booster mRNA)	33	15 (45.5%)	0.78 (0.51-1.17)	0.239	0.44 (0.28-0.69)	<0.001
**The household contacts' COVID-19 vaccination x time**				<0.001		<0.001
Not vaccinated, vaccinated 1 dose≤21 days	102	62 (60.8%)	1.00		1.00	
Vaccinated 1 dose>21 days or 2 doses≤7 days	72	31 (43.1%)	0.70 (052.-0.96)	0.028	0.73 (0.53-1.02)	0.066
Vaccinated 2 doses 8-90 days	73	27 (37.0%)	0.60 (0.43-0.85)	0.004	0.47 (0.33-0.68)	<0.001
Vaccinated 2 doses>90 days or 3 doses≤7 days	142	83 (58.5%)	0.95 (0.77-1.16)	0.679	0.66 (0.51-0.86)	0.002
Vaccinated 3 doses 8-151 days	73	26 (35.6%)	0.58 (0.41-0.82)	0.002	0.30 (0.20-0.45)	<0.001

[a] Generalized Estimating Equations using negative binomial models, yielding relative risk (RR), 95% confidence intervals (CI) and P values..

COVID-19: RT-PCR confirmed SARS-CoV-2 infection in at least one follow-up nasal swab plus symptoms (at least one), 9 individuals with asymptomatic infection were excluded from the analysis. The results of the covariates relate to model of in which COVID-19 vaccination status of was introduced as the number of doses. The results for the covariates in the remaining models that related to 'vaccine brand' and number of doses by time elapsed since vaccination' were similar and thus not presented

ChAdOx1-S: the adenovirus vector vaccine (Oxford-AstraZeneca) COVID-19 vaccine, mRNA vaccine: either mRNA-1273 (Moderna, Inc.) or BNT162b2 (Pfizer-BioNTech) vaccines.

Supplementary Table S8. The associations of COVID-19 vaccination with overall SARS-CoV-2 infection and COVID-19 among household contacts aged 20 years or older- sensitivity analyses.

	**Overall infection [a,b]**		COVID-19 [b,c]	
	**Adjusted RR (95% CI) [e]**	P value	Adjusted RR (95% CI) [e]	P value
**COVID-19 vaccination of the household contact**		<0.001		<0.001
Not vaccinated	1.00		1.00	
1 dose	0.80 (0.57-1.10)	0.174	0.80 (0.57-1.12)	0.197
2 doses	0.62 (0.46-0.84)	0.002	0.61 (0.45-0.83)	0.002
3 doses	0.33 (0.22-0.50)	<0.001	0.30 (0.20-0.45)	<0.001
**The household contacts' COVID-19 vaccination x Brand**		<0.001		<0.001
Not vaccinated	1.00		1.00	
1 dose ChAdOx1-S	0.97 (0.66-1.43)	0.902	0.97 (0.65-1.45)	0.894
1 dose mRNA vaccine	0.61 (0.37-1.02)	0.061	0.63 (0.37-1.05)	0.080
2 doses ChAdOx1-S	0.72 (0.52-1.00)	0.050	0.71 (0.51-0.99)	0.046
2 doses mRNA vaccine	0.54 (0.38-0.77)	0.001	0.53 (0.37-0.76)	0.001
3 doses mRNA vaccine	0.31 (0.19-0.50)	<0.001	0.25 (0.15-0.43)	<0.001
3 doses (2 ChAdOx1-S booster mRNA)	0.38 (0.23-0.61)	<0.001	0.36 (0.22-0.59)	<0.001
**The household contacts' COVID-19 vaccination x time**		<0.001		<0.001
Not vaccinated, vaccinated 1 dose≤21 days	1.00		1.00	
Vaccinated 1 dose>21 days or 2 doses≤7 days	0.69 (0.49-0.96)	0.028	0.71 (0.51-1.00)	0.055
Vaccinated 2 doses 8-90 days	0.48 (0.33-0.72)	<0.001	0.48 (0.32-0.71)	<0.001
Vaccinated 2 doses>90 days or 3 doses≤7 days	0.71 (0.51-0.98)	0.041	0.70 (0.50-0.98)	0.041
Vaccinated 3 doses 8-151 days	0.29 (0.19-0.45)	<0.001	0.25 (0.16-0.39)	<0.001

[a] Overall infection: RT-PCR confirmed SARS-CoV-2 infection in at least one follow-up nasal swab.[b] The model included calendar month-year to adjust for variant/period [c] COVID-19: RT-PCR confirmed SARS-CoV-2 infection in at least one follow-up nasal swab plus symptoms (at least one); asymptomatic individuals were excluded from the analysis [d] Generalized Estimating Equations using negative binomial models yielding relative risk (RR), 95% confidence intervals (CI) and P values: each model included age, sex, household size, vaccination status, and time variable (calendar month-year) as independent variables. ChAdOx1-S: the adenovirus vector vaccine (Oxford-AstraZeneca) COVID-19 vaccine, mRNA vaccine: either mRNA-1273 (Moderna, Inc.) or BNT162b2 (Pfizer-BioNTech) vaccines

Supplementary Table S9. COVID-19 vaccination status of household contacts aged 20 years or older by variant period.

	Alpha	Delta	Omicron
	Weeks 6-18, 2021	Weeks 19-48, 2021	Weeks 49-52 2021 & 1-6, 2022
Total	126 (100.0%)	270 (100.0%)	75 (100.0%)
Not vaccinated	57 (45.2%)	29 (10.7%)	7 (9.3%)
1 dose≤21 days	9 (7.1%)	3 (1.1%)	0 (0.0%)
1 dose>21 days	51 (40.5%)	18 (6.7%)	0 (0.0%)
2 doses≤7 days	1 (0.8%)	2 (0.7%)	0 (0.0%)
2 doses 8-90 days	8 (6.3%)	65 (24.1%)	1 (1.3%)
2 doses>90 days	0 (0.0%)	121 (44.8%)	19 (25.3%)
3 doses≤7 days	0 (0.0%)	3 (1.1%)	1 (1.3%)
3 doses 8-151 days	0 (0.0%)	29 (10.7%)	47 (62.7%)

Supplementary Table S10. The associations of COVID-19 vaccination with overall SARS-CoV-2 infection among household contacts aged 20 years or older during the Alpha variant period (N=126) -a sensitivity analysis [a].

The contact's vaccination status	n/Total (%)	Unadjusted RR (95% CI) [b]	P value	Adjusted RR (95% CI) [c]	P value
**COVID-19 vaccination**			0.015		0.025
Not vaccinated	34/57 (59.6%)	1.00		1.00	
1-2 doses	26/70 (37.1%)	0.63 (0.43-0.91)		0.59 (0.37-0.93)	
**COVID-19 vaccination x time**			0.007		0.001
Not vaccinated, vaccinated 1 dose≤21 days	41/66 (62.1%)	1.00		1.00	
Vaccinated 1 dose>21 days or 2 doses≤7 days	17/52 (32.7%)	0.52 (0.34-0.81)	0.004	0.44 (0.27-0.73)	0.001
Vaccinated 2 doses 8-90 days	2/8 (25.0%)	0.40 (0.11-1.35)	0.142	0.28 (0.08-0.99)	0.048

[a] Overall infection: RT-PCR confirmed SARS-CoV-2 infection in at least one follow-up nasal swab [b] Bivariate Generalized Estimating Equations using negative binomial models, yielding relative risk (RR), 95% confidence intervals (CI) and P values [c] Generalized Estimating Equations using negative binomial models: each model included in addition to the contact's vaccination status the following independent variables: age, sex, household size, and time-variable as calendar month/year to adjust for variant/period.

Supplementary Table S11. The associations of COVID-19 vaccination with overall SARS-CoV-2 infection among household contacts aged 20 years or older during the Delta variant period (N=270) [a].

The contact's vaccination status	n/Total (%)	Unadjusted RR (95% CI) [b]	P value	Adjusted RR (95% CI) [c]	P value
**COVID-19 vaccination**			0.004		<0.001
Not vaccinated	17/29 (58.6%)	1.00		1.00	
1 dose	15/21 (71.4%)	1.21 (0.81-1.83)	0.343	1.12 (0.68-1.86)	
2 doses	97/187 (51.9%)	0.88 (0.62-1.23)	0.458	0.56 (0.36-0.87)	0.011
3 doses	6/33 (18.2%)	0.31 (0.14-0.68)	0.003	0.17 (0.07-0.40)	<0.001
**COVID-19 vaccination x time**			<0.001		0.001
Not vaccinated or vaccinated 1 dose≤21 days	20/32 (62.5%)	1.00		1.00	
Vaccinated 1 dose>21 days or 2 doses≤7 days	14/20 (70.0%)	1.12 (0.75-1.65)	0.572	1.06 (0.67-1.69)	0.789
Vaccinated 2 doses 8-90 days	26/65 (40.0%)	0.64 (0.42-0.95)	0.029	0.50 (0.31-0.87)	0.005
Vaccinated 2 doses>90 days or 3 doses≤7 days	72/124 (58.1%)	0.92 (0.67-1.25)	0.613	0.56 (0.36-0.87)	0.011
Vaccinated 3 doses≥8 days	3/29 (10.3%)	0.16 (0.05-0.50)	0.001	0.10 (0.03-0.32)	<0.001

[a] Overall infection: RT-PCR confirmed SARS-CoV-2 infection in at least one follow-up nasal swab [b] Bivariate Generalized Estimating Equations using negative binomial models, yielding relative risk (RR), 95% confidence intervals (CI) and P values. [c] Generalized Estimating Equations using negative binomial models: each model included age, sex, household size, vaccination status, and time-variable calendar month to adjust for variant/period as independent variables.

Supplementary Table S12. The associations of COVID-19 vaccination with overall SARS-CoV-2 infection among household contacts aged 20 years or older during the Omicron variant period (N=75) [a].

The contact's vaccination status	n/Total (%)	Unadjusted RR (95% CI) [b]	P value	Adjusted RR (95% CI) [c]	P value
**COVID-19 vaccination of the contact**			0.643		0.048
Not vaccinated, vaccinated 2 doses or 3 doses ≤7 days [d]	17/28 (60.7%)	1.00		1.00	
3 doses 8-151 days	26/47 (55.3%)	0.91 (0.61-1.35)		0.65 (0.42-0.99)	

[a] Overall infection: RT-PCR confirmed SARS-CoV-2 infection in at least one follow-up nasal swab. [b] Bivariate Generalized Estimating Equations using negative binomial models, yielding relative risk (RR), 95% confidence intervals (CI), and P values. [c] Generalized Estimating Equations using a negative binomial model, which included the independent variables: age, sex, household size, the contact's vaccination status, and time-variable calendar month/year to adjust for variant/period as independent variables. [d] Unvaccinated participants, those who were vaccinated with two doses (regardless of time elapsed since vaccination) or three doses ≤seven days, were grouped into one category.

Supplementary Table S13. The correlation between the number of COVID-19 vaccination received by the index cases and their household contacts.

		Number of COVID-19 vaccines doses received by the contact				Total	Spearman's correlation coefficient	P value
		**0**	**1**	**2**	**3**			
**Number of COVID-19 vaccines doses received by the index case**	**0**	**42**	**11**	16	2	71	0.72	<0.001
	**1**	35	48	9	0	92		
	**2**	15	23	161	15	214		
	**3**	1	0	31	63	95		
Total		93	82	217	80	472		

Supplementary Table S14. The association of COVID-19 vaccination and Ct values of the E gene of SARS-CoV-2 positive participants on their first positive swab – a pooled analysis of index cases and household contacts.

	Number	Ct value≤24.93 (median)	Ct value>24.93 (median)	Adjusted RR (95% CI)[a]	P value
Not vaccinated	119	69 (58.0%)	50 (42.0%)	1.00	
1 dose	112	66 (58.9%)	46 (41.1%)	0.98 (0.89-1.08)	0.754
2 doses	297	152 (51.2%)	145 (48.8%)	1.05 (0.97-1.15)	0.201
3 doses	109	44 (40.4%)	65 (59.6%)	1.11 (1.00-1.24)	0.038

[a] Generalized Estimating Equations using negative binomial models, yielding relative risk (RR), 95% confidence intervals (CI) and P values. The model included Ct values as the dependent variable, comparing high Ct values (above the median) to lower Ct values. The independent variable was vaccination status, the model adjusted for case/contact status and variant periods.
